# Social and health care top managers’ perceptions and aims of strategic work ability management in the midst of change

**DOI:** 10.3233/WOR-230034

**Published:** 2024-02-07

**Authors:** Julia Anttilainen, Irmeli Pehkonen, Minna Savinainen, Eija Haukka

**Affiliations:** a Finnish Institute of Occupational Health, Helsinki, Finland; b Varma Mutual Pension Insurance Company, Helsinki, Finland

**Keywords:** Leadership, occupational health, health promotion, disease management, change management, human resources

## Abstract

**BACKGROUND:**

Existing research has mostly focused on themes related to returning to or staying at work and studied organizations’ operational rather than strategic level actions to support work ability. Top managers’ understanding of work ability management (WAM) may influence how work ability support processes are implemented in organizations.

**OBJECTIVE:**

To find out how top managers define WAM, what the aims of WAM were and whether the aims were on a strategic level.

**METHODS:**

Altogether 28 semi-structured interviews among Finnish social and health care top managers were conducted during the years 2019–2021 and analyzed inductively using qualitative thematic analysis.

**RESULTS:**

Top managers’ definition of WAM was mainly multidimensional. Two main aims were identified, i.e., to support work ability 1) at the individual and 2) at the organizational level. The aims of the former were to anticipate the decrease of health and functional capacity, to support workers already decrease in these, to develop competence, and to manage the effects of changes on work ability. The aims at the organizational level were to improve labor availability and personnel retention, to ensure the flow of work, and to increase trust and create shared values. Top managers described the aims as being at a strategic level, but this was not yet realized in their organizations because the actions were reactive rather than proactive.

**CONCLUSION:**

Top managers’ multidimensional perception of WAM, emphasizing proactive actions, and strategic level aims are crucial and require the commitment of the top managers for strategic WAM, especially during constant changes.

## 1 Introduction

### 1.1 Work (dis)ability management

Work ability as a concept is essential for many sciences and actors related to working life. The origins of the commonly used work ability model are in the early 1980’s in Finland, when there was a social need for a new, positive approach [1]. However, there is no unified interpretation that is unanimously accepted by, e.g., insurance and rehabilitation institutions, occupational health care, or researchers representing different disciplines [2, 3]. Although there is no single definition, there exists a consensus that work ability is defined by one’s work, work environment and societal context [1, 3–5].

Regarding management of work ability, the international literature usually refers to the concept of “work disability” and “ work disability management”, instead of the “work ability” point of view. Over the past 30 years, research has mostly focused on themes related to organizational challenges for the employer, such as facilitating a return-to-work after the acute onset of illness or injury, enabling staying-at-work for workers with chronic conditions, decreased work ability or recurring symptoms, and providing effective workplace accommodations and support for workers with disabilities [6]. Work disability management has proven to be effective in reducing sick leave, increasing retirement age, and reducing the costs of disability [7–11]. However, the previous literature has usually focused on individual rather than organizational level actions [12] and organizations’ operational rather than strategic level actions.

### 1.2 Strategic work ability management

To our knowledge, there is no previous research on strategic work ability management (WAM) and it does not have a general definition. Therefore, we refer to strategic management, which is an approach to identify and address challenges. It is a set of concepts, processes, procedures, tools, techniques, structures, and practices that must be drawn on selectively and adapted thoughtfully to specific contexts to be able to help produce desirable results. Strategizing means merging goals and capabilities, thus making sure that goals can be achieved [13]. Top managers are the decision makers and create knowledge and values to the decision-making process [14–16].

According to Jasper and Crossan [17], the features of strategic management are 1) the process of organizational leadership, recognizing and involving the whole workforce; 2) envisioning and responding to the future through fostering anticipation; 3) providing processes for coping with change and organizational development, with a focus on performance and achieving organizational strategic objectives; and 4) facilitating and communicating consistent decision-making. Thus, strategic management requires goals [13, 17–19] and strategy itself links together capabilities and objectives of the organizations and thus helps to achieve the goals [13].

While strategic management has developed across disciplinary boundaries, little has yet been reported of its specific nature in healthcare management theoretical perspectives [17] or on how organizations can and wish to implement strategies to promote a healthy and sustainable working life [20].

### 1.3 Background of the study

In Finland, the public health and social service sector has been under nationwide reorganization and changes for many years. The municipalities and hospital districts have been responsible for organizing health and social services. From the year 2023 onwards, the responsibility will be on larger wellbeing services counties [21]. These kinds of reforms may require changes in organizations’ values, structures, and processes [22]. This study is based on the “Strategic work ability management in the social and health care reform (2019–2022)” project. The aim of the project was that health and social care organizations adopt WAM as part of strategic management, which creates the prerequisites, operating methods, and monitoring tools for maintaining and developing the personnel’s work ability in changes [23, 24].

In the above-mentioned project and in this study, we have defined work ability from a multidisciplinary point of view including employees’ competence, motivation, values, health, and functional capacity [1]. WAM includes all activities that are planned and followed in the organization itself and in cooperation with other specialists, such as occupational health care providers, to promote and maintain occupational safety, work ability and health, and to support staying at work [25]. Too often, the disability point of view may have taken the focus away from work ability as a resource. This may be because to obtain disability compensation, it is obligated to demonstrate the severity of disability [26]. Concerns about labor availability and aging workforces, the aim to control social and health care costs and extending working careers, understanding how to develop and maintain employees’ work ability is even more important [4, 22].

To our understanding, for WAM to be strategic, it must be part of daily management and the organization’s strategy, vision, and values, and it should be done in collaboration with occupational health care service, other specialists, rehabilitation, social insurance, and authorities. Currently it has also been highlighted that workplaces should integrate WAM into their management practices [27].

Top management is responsible for the organization’s strategy, including human resource management [14] and WAM as part of it. We aim to support and strengthen the concept of work ability instead of the concept of work disability. Our research contributes to the previous findings that the way top management define and understand WAM may influence measures and processes aimed to support personnel’s work ability and how these are implemented in the organizations [3, 4, 28]. In addition, top managers’ perceptions have an impact on organizations’ strategic choices and success [15]. As far as we are aware, there is a lack of studies concerning WAM from this point of view. In this paper, our aim was to find out how top managers define WAM, what were their aims for WAM, and whether the aims were on a strategic level.

## 2 Material and methods

### 2.1 Data collection

The project on which this research is based was carried out in five large Finnish joint municipal authorities for social and health care organizations. As part of the project, interview data were collected, e.g., to get an understanding about the state of WAM and to support the development work. The current research utilizes top management’s semi-structured interviews, which were conducted during June and October 2019, January 2020, and September 2021.

According to Graebner et al. [29], qualitative methods are useful for generating theory when the phenomenon being studied is new or there are no previous studies. Qualitative method and phenomenological approach were chosen to enable interviewees to discuss their own experiences and interpretations [30, 31]. We considered it important to focus on top managers as key stakeholders to facilitate the strategy and decide about the resources. To achieve credibility, it is crucial to find participants who probably have experiences of the phenomenon under study [32]. Inclusion criteria for the participants were that they had to be a member of their organization’s executive team. The interviewees were found with the help of project actors, such as work ability coordinators working in the joint municipal authorities for health and social services, who provided a list of their organizations’ top managers. The interviewees were contacted and invited to the interview by email which included an information letter about the current research and the project behind the study. No one refused to participate in the interviews. Out of a total of 16 interviews, 14 were conducted face to face at the interviewees’ workplace, and due to the COVID19-pandemic, the last 2 via remote connection. No other persons were present besides the interviewers and the interviewees. The interviewed top managers represented the Finnish joint municipal authorities for social welfare and healthcare from five different geographical areas and different sectors (e.g., services for the disabled or for the elderly). All participants had long experience in the social and health care sector. The data consists of 10 group and 6 individual interviews in Finnish. The total number of interviewees was 28 (18 females and 10 men) (Table 1).

**Table 1:** Interviewees in the Finnish joint municipal authorities for social and health care

Interview	Top manager (TM)	Organization	Sex^a^
Individual	01	1	M
Group	02	1	M
03	1	F
04	1	M
Group	05	1	F
06	1	F
07	1	F
08	1	M
Group	09	2	F
Group	10	2	M
11	2	M
Group	12	2	F
13	2	F
Group	14	2	F
15	2	F
Individual	16	2	F
Individual	17	3	M
Individual	18	3	M
Group	19	3	M
20	3	M
Individual^b^	21	4	F
Individual^b^	22	4	F
Group	23	5	F
24	5	F
Group	25	5	F
Group	26	5	F
27	5	F
28	5	F
**Altogether**	**28**	**5**	**18 F / 10 M**
**Individual**	**6**		
**Group interview**	**10**		

^a^F = Female, M = Male. ^b^Conducted online.

### 2.2 Interviews

All interviews were conducted by two researchers of the project group, at least one of which was an expert in WAM or in qualitative research and had a PhD degree. The interviews were conducted by the first author (female, MSc, researcher), the second author (female, PhD, senior specialist), senior specialist (female, PhD) and specialist Doctor of Occupational Health (male, DMedSci) from Finnish Institute of Occupational Health (FIOH).

There were no relationships established between the researchers and the interviewees before the study. The interviewees had no prior knowledge of the researchers and they introduced themselves in the beginning of the interviews (name and position at the FIOH). The researchers had previous knowledge about WAM and its successful implementation, but no previous knowledge about the interviewees and their perceptions of WAM. The interviewers did not identify prior assumptions or biases that would have influenced the interviews.

The research questions were guided by the objectives of the core project and previous research on successful WAM [9, 25]. The interviews were conducted using a semi-structured protocol. Interview themes focused on the importance of WAM and perceptions of its strategy (e.g., is WAM considered to be on a strategic level), processes (e.g., what practices are used in supporting work ability), roles, responsibilities (e.g., top managers’ own role in WAM), leading with knowledge (e.g., whether the organization collects anticipative data about work ability) and collaboration (e.g., between organization and occupational health care). All participants were asked follow-up questions for clarification when necessary. No questions were provided in advance, no pilot testing conducted, and no repeated interviews were carried out.

The interviews were audio-recorded and transcribed verbatim. The transcriptions were not returned to the participants. No field notes were written during the interviews. The duration of the interviews varied from 43 minutes to 1 hour 23 minutes. Two members of the research team listened to each interview, verified all transcribed data for accuracy and pseudonymized the transcribed data to protect confidentiality.

### 2.3 Analysis

The familiarity required for the thematic coding was ensured by multiple readings of the transcripts in an active way searching for meanings and patterns [33, 34]. The analysis proceeded following the six-step framework by Braun and Clarke [33], starting with reading through the transcribed data (Table 2). To understand the top managers’ perceptions and the aims of WAM, an inductive thematic analysis was used. The strategy of inductive analysis does not presuppose in advance what are the relevant dimensions in the data and the researcher allows them to emerge from the patterns found in the cases under study. Inductive analysis begins with specific observation and builds toward general patterns [30].

**Table 2:** Six phases of thematic analysis procedure (Braun & Clarke 2006)

Phase	Examples
1. Familiarization with the data	Listening, reading, re-reading, making notes of potential interest or ideas to explore further, reflection on similarities and differences.
2. Coding the data	Creating initial codes and coding analytically interesting and relevant data. Peer debriefing.
3. Generating initial themes	Organizing the codes into initial themes. Gathering all coded data relevant to each theme.
4. Reviewing and developing themes	Checking if the themes work in relation to the coded extracts and the entire data set. Finalizing thematic map.
5. Refining, defining, and naming themes	Continuous analysis with the research team to refine the specifics of each theme.
6. Producing the report	Analyzing analytic commentary, data extracts and themes. Discussing the analysis and selecting examples of data to illustrate each theme. Relating analysis to research questions and literature. Writing the report.

The transcribed data were downloaded to Atlas.ti software program (version 9) which was used to manage the data and for coding. The data was categorized and named inductively while reading. The aim was to identify patterns in the data that are interesting in relation to the research question [35]. All researchers participated in the first steps of the coding process to familiarize themselves with the data. The researchers conducted reflective writing in Atlas.ti -program using memos and comment tools while coding the data. In addition, they wrote down thoughts about what the coded data extracts mean and how they interpret them. This supported the next stages of the analysis and the trustworthiness of the study [34, 36]. Thereafter, the first author performed coding of the whole data. To establish trustworthiness and address dependability [30, 32], the codes and themes derived from the data were discussed in the meetings within the research group (peer debriefing). The researchers reviewed the coded data extracts and sub-themes to gain mutual understanding. Disagreements in the data analysis were resolved and changes made, if necessary, for example if a sub-theme did not have enough data to support the theme [34]. The participants were not provided feedback on the findings.

## 3 Results

### 3.1 Top managers’ definitions of WAM

The top managers considered WAM an important issue, part of daily management, and it should be understood more comprehensively in their organizations instead of approaching it via a narrow concept of work ability. In some organizations WAM was discussed using different concepts, such as competence or occupational health management. WAM was also defined through the concept of work ability. WAM was understood as being not only individual measures, but also proactive actions, part of good management of the organization, and that all actors have responsibility for it.

*Top manager (TM) 07:* “*I see it as part of the strategy and being implemented in the strategy...All of the employees*’ *activities related to competence, coping, well-being at work and training are associated with it.*”

*TM19:* “*It is an extensive package, which includes all factors that affect a person*’*s work ability* – *how they have been modelled and taken into account* – *and they are the factors that also affect the management. The management should have an understanding and view of all the different factors that contribute to people being able to work and retain their work ability.*”

*TM05:* “*Describes mainly the activities of the employer or HR, but we all also have the responsibility to manage our own work ability and ensure that we do not endanger our own work ability or that of colleagues.*”

The strategic nature of WAM was seen to require systematicity, clear goals, and that it should be measured and followed. However, the organizations were not yet at this level, and the interviewees described that WAM is still in its infancy and has not necessarily been included in the organization’s strategy. Some of the interviewees mentioned that, especially during organizational changes, there is a lot of discussion related to WAM at the executive board level.

*TM14:* “*It must be derived from our strategy, so it is a familiar term. It must be a shared matter that not only concerns the individual but also the entire work community, even though it may be visible at the individual level.*”

*TM06:* “*Work ability management means that we have a clear strategy, vision, mission and goals, responsibilities, and roles, and, nevertheless, we have this shared [Organization 1] thing. It shouldn*’*t be this kind of ambiguous thing, meaning that everyone is just bouncing around wondering what they could do.*”

### 3.2 Top managers’ aims of WAM

**Fig. 1:**
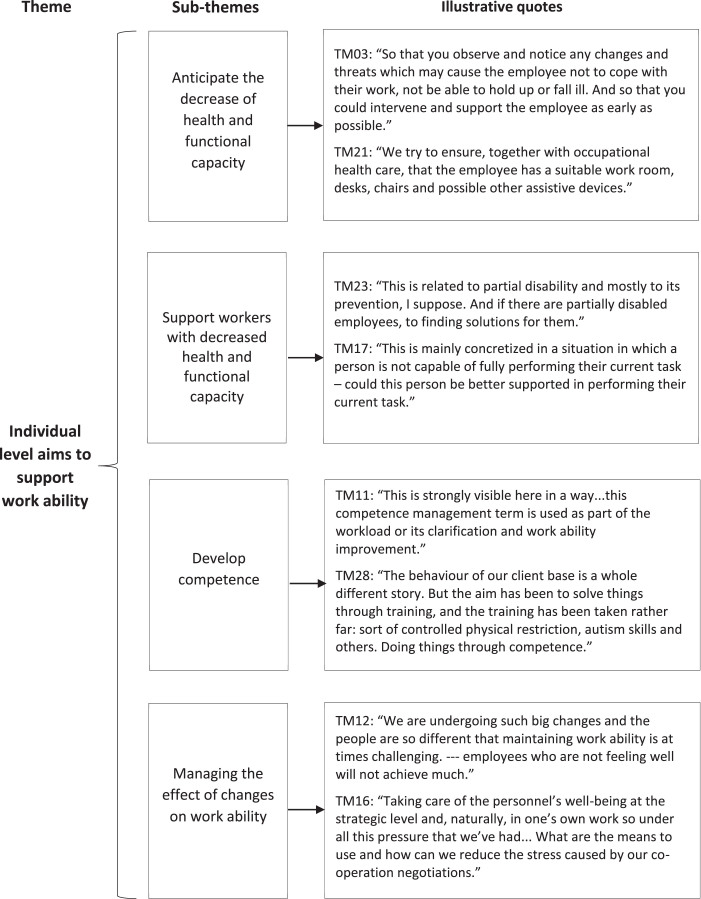
Top managers’ aims of WAM at the individual level with illustrative quotes.

**Fig. 2:**
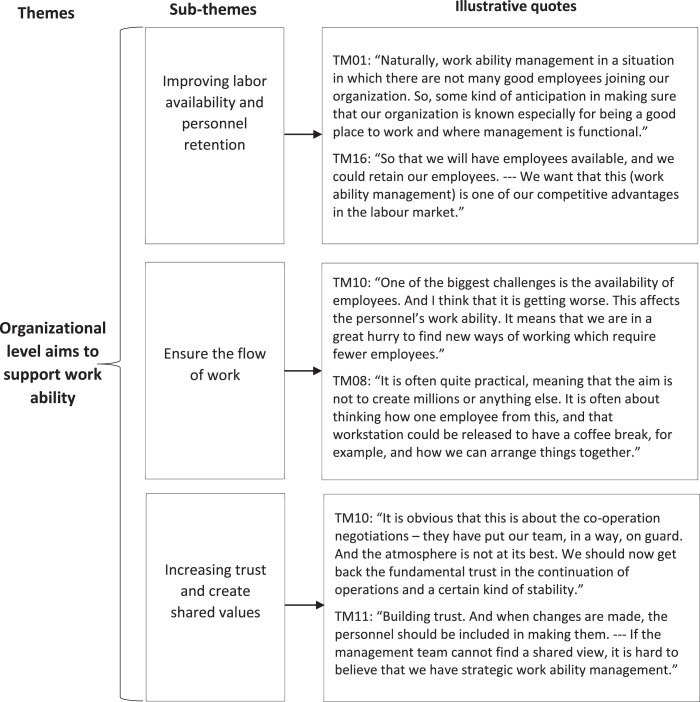
Top managers’ aims of WAM at the organizational level with illustrative quotes.

Two main and seven sub-themes were identified to describe what top managers aim for with WAM. Firstly, WAM was understood as managing and supporting work ability at the individual level, and secondly, at the organizational level. In the following, we present the results concerning the main themes followed by the subthemes. Examples of citations are presented in Figs. 1 and 2.

#### 3.2.1 Individual level aims to support work ability

*Anticipate the decrease of health and functional capacity* The top managers highlighted that WAM should be implemented in an anticipatory way, but measures supporting work ability are often undertaken too late in organizations. The aim of WAM was seen to detect possible threats that influence the individual employees’ work ability, or well-being of the whole working community. One interviewee stated that in the future, those employers who can secure their employees’ work ability will be successful.

WAM meant also taking work ability into account in all decision-making. According to this, all measures and decision-making aim to support the maintenance, improvement, or development of work ability. However, some interviewees reminded that also individuals have responsibility for maintaining their own work ability while with organizations’ WAM practices it is not possible to manage everything.

Supporting the existing work ability and thus also influencing the performance and results of the whole team was considered important. In some organizations WAM was aimed at reducing absenteeism, e.g., by systematically developing risk management or organizing targeted small group activities to support employees’ physical condition.

One interviewee mentioned collaboration with occupational health service provider to ensure suitable work equipment and work environment. The organization’s operating models aim to catch and support the first signs of a weakening of resources as early as possible. In daily management, the aim is to either strengthen the employee’s ability to work or, for example, to be temporarily or permanently reassigned to different tasks.

*Support workers with decreased health and functional capacity* In general, WAM was described as reactions in cases where there already were challenges in work ability. Work ability was managed on a case-by-case basis and reacted to if needed. In a stable situation, not much attention was paid to practices supporting work ability. One interviewee even connected WAM solely supporting workers with partial work ability and finding solutions to support staying at work.

The interviewees commented that the amount of sickness absences activate organizations to actions to support workers work ability. However, if the written goal in the organization was to reduce sickness absences, it was not always reached. Further, the actions were taken only in case of an increase in sickness absences. One interviewee stated that by reducing sickness absences, the financial savings needed by the organization could be achieved.

*Competence development* Competence development was seen as part of WAM, because deficiencies in competence increase workload. For example, in social work management, it was aimed to ensure both the skills of employees and the capability of the work community. Some of the top managers considered that WAM was a means to develop competence, and that way manage dangerous situations in customer services. Also, taking care of personnel’s competence by WAM was seen as important in developing a sustainable organization and enabling individual career development.

*Managing the effect of changes on work ability* The ongoing changes, tightening economy, and lack of resources in the organizations were in the background influencing top managers’ definitions and the aims of WAM. The increasing demands created pressure for employees’ work ability. Well-being personnel was considered to adapt better to changes, and thereby the top management aimed to manage the effects of changes on work ability. The personnel’s professional skills and good management were considered to strengthen work ability and to be a prerequisite for implementing changes. Good management would also prevent personnel from being overburdened during changes. Some top managers stated that WAM should be included as part of strategic management to be able to better respond to the personnel shortage. Strengthening the personnel’s resilience and well-being at work were part of the strategic aims.

Good communication was seen to support employees and the work community during changes. The aim of WAM was to proactively support work ability by increasing dialogue and interaction. Especially, co-determination negotiations caused major challenges in the organizations. Some top managers aimed to influence the stress caused by the negotiations, e.g., by supporting the supervisors or the entire work community in dismissals.

#### 3.2.2 Organizational level aims to support work ability

*Improving labor availability and personnel retention* Labor shortage is a significant challenge in the social and health care sector. Top managers stated that good WAM increases competitive advantages in the labor market and may influence the organization’s success and inspire confidence. Therefore, the aim of WAM was to influence the employer brand – labor availability and personnel retention. Some top managers underlined that it is essential to invest in WAM, because the labor shortage is expected to get worse. Planning how to commit competent personnel to the organization would be essential, but this is not currently done.

*Ensure the flow of work* The aim of WAM was seen to ensure that everyone’s job description was clear, they were motivated, and work tasks were suitable for them. In daily WAM, financial aims were not the focus. Instead, WAM was seen more as practical, where the aim was to find solutions to secure work processes despite tightening resources. Co-determination negotiations will force organizations to limit and focus on the main tasks even more precisely.

Due to the decrease in resources, new ways of working were needed. Some top managers mentioned that the changes and the labor shortage also affect productivity. In coming years, the supply of social and health care services was not seen to be able to meet demands. Top managers aimed to manage the stress of personnel by developing processes and guiding customers effectively to the right services. Top managers emphasized personnel participation in finding solutions.

*Increasing trust and create shared values* Constant changes in the organizations had caused uncertainty and mistrust among employees. WAM was seen as one of the key factors to create shared values, operating models, and rules to increase mutual trust in the future. Workers’ engagement was seen as important to achieve team spirit and common values.

In one organization, the strategic level aim was that management is confidence-inspiring and appreciative of personnel. The role of top managers was seen to act as an example and show that work ability is important, and measures related to WAM are in accordance with the organization’s values. The interviewees described that it is difficult to implement strategic WAM if the top managers do not share the same aims and pull together.

## 4 Discussion

Our aim was to find out how Finnish top managers representing social and health care define WAM, what were their aims of WAM, and whether the aims were on a strategic level. The definitions of WAM were mainly multidimensional, which could also be seen in the aims of WAM. The ongoing changes also determined the way the top managers discussed the aims of WAM. We identified two main themes describing the aims: Managing and supporting work ability at 1) individual and 2) organizational level. This is in line with Lederer et al. [3] on recognizing that work ability is multidimensional, and both individual and organizational issues have important implications for employers in terms of work (dis)ability promotion, prevention, rehabilitation, and management. This enlarged vision of work ability may enable innovative ways to utilize solutions to support worker’s continuing at work and return to work [3]. In our opinion, this implies also anticipative actions. This point of view has also been emphasized in the previous literature [1, 3, 37, 38]. However, the responsibilities of different stakeholders in WAM are not discussed enough. More understanding is needed about WAM as part of organizations’ human resource strategies and the role of top managers.

The definition of WAM may be influenced by the top managers’ perceptions of work ability. The interviewees who were not familiar with WAM as a concept defined it through the concept of work ability. The perception of work ability may affect how work ability support processes are implemented [3, 4, 28]. Therefore, it is important that different stakeholders in organizations have extensive and shared understanding of issues affecting work ability.

According to the multidimensional concept map of work (dis)ability by Lederer et al. [3], individual level includes all dimensions of work ability related to the employee’s condition. This must be understood as encompassing all personal assets, facilitators and barriers which characterize the worker, such as skills, health, and values. The organizational level considers the organizational factors that contribute to shaping or structuring work ability, including attributes related to, e.g., physical, and social work environment. Similarly, based on our findings, top managers seemed to emphasize both the individual and organizational level. Contrary to Lederer et al. [3], we interpreted values at the organizational level because the top managers aimed to create shared organizational values.

### 4.1 Individual level aims to support work ability

The individual level aims of WAM were anticipating the decrease of health and functional capacity, supporting workers with decreased health and functional capacity, developing competence, and managing the effect of changes on work ability. Only one interviewee, interviewed individually, considered WAM as solely reacting to changes in work ability, others saw also the anticipative dimensions of WAM. One interviewee participating in a group interview also saw WAM mainly as competence management. Although the interviewees themselves considered anticipation to be important, the common goal in the organizations seemed to be the decrease in sickness absences. This is in accordance with previous research where actions often target work disability [6].

Top managers’ own understanding of what is or should be aimed for with WAM may differ from the organization’s written goals [39], as was observed also in our study. The top managers’ understanding of the importance of proactive actions in supporting work ability is encouraging and hopefully will guide the strategic aims of the organization in the future. In the social and health care sector personnel’s workload has been much discussed, and managers may be more aware of the importance of WAM than in other industries.

Positively, top managers included competence development as part of WAM. Especially, in the constantly changing work environment also competence demands may change quickly. If the top managers did not think competence management through organizational measures important, it could be difficult for employees to cope with the changes [38]. On the other hand, limited resources may be an obstacle to offer possibilities to develop professional skills [40].

### 4.2 Organizational level aims to support work ability

The organizational level aims of WAM were improving labor availability and personnel retention, ensuring the flow of work, and increasing trust and creating shared values. The ongoing changes seemed to affect especially the aims at an organizational level. Constant changes had caused uncertainty and mistrust among personnel and the top managers emphasized the importance of bringing back confidence. Also, Jasper and Crossan [17] have noticed that the cultural beliefs and values of personnel have an impact on the development and implementation of the successful strategy. We found that the labor shortage had increased workload and WAM was recognized as a means to ensure sufficient personnel resources. This is in accordance with the findings of Eriksson et al. [41] on the importance of the health promoting leadership as a part of developing an attractive workplace.

Managers aimed to secure employees’ capability to provide high quality services despite the ongoing changes and lack of personnel. This could refer to top managers consideration of employees being not only a resource, but a prerequisite for the organization’s success. Productivity was also the main issue as an outcome of work ability in the study of Jansson et al. [28]. The employees’ work ability has been regarded as a tool in production and not a goal itself [28, 41].

The prevailing labor shortage in the field seemed to reflect what top managers aimed for with WAM on the organizational level. This is a significant and new perspective which separates WAM from work disability management that usually targets individual level actions [6]. According to previous studies, individual level actions alone are not effective when supporting work ability [12]. In a systematic review by Montano et al. [42], the best results were achieved by multicomponent interventions.

### 4.3 Strategic nature of the aims

In general, WAM was strongly emphasized as part of the organization’s strategy and considered important. However, WAM was not yet visible in the organizations at a strategic level or WAM wasn’t even included in the strategy. As was seen throughout the interviews, constant changes affected the discussion about WAM. Too frequent re-organizations have been regarded as an obstacle and could have a negative impact on the development of sustainable structures supporting health promoting leadership [22, 41]. Despite the tightening economy and commonly known high levels of sickness absence in social and health care sector, the pursuit of financial savings was not emphasized. This was interesting while in a review by Figueroa et al. [22] noticed that political decisions often focus on cost savings resulting financial resource constraints in the health services’ operating environment. These constraints may lead to poor human resource management and create an imbalance between service demand and supply [22]. The availability of employees and securing the flow of work were considered more important. This could indicate that for the top managers WAM is strongly related to the organizations’ values, like in the upper echelons theory (UET), in which the values of top managers’ may influence the strategic decisions [14–16].

Referring to Jasper and Crossan [17], the top managers’ aims of WAM seemed to be on a strategic level. Personnel participation, which is one of the key features of strategic management, was seen important in the midst of changes. The top managers aimed to anticipate future personnel needs. Accordingly, top managers were worried about the coping of the personnel during changes and providing processes for it. Jasper and Crossan [17] also emphasized facilitating and communicating consistent decision-making. However, this did not emerge in our data. Since top managers have an important role in facilitating goal attainment and visions of the organization and enabling personnel engagement [43], more attention should be paid to discussing the strategic aims of WAM.

This study was conducted during major changes in the social and health care sector. Regardless of the industry, both global and local changes affect working life. Therefore, WAM should always be acknowledged and considered that every decision made affects personnel’s work ability. We viewed only the top managers’ aims and did not compare them to the organizations’ written aims. The two being aligned would be essential for the implementation of the strategy. The presented findings could be useful when developing organizations’ strategic WAM.

### 4.4 Strengths and limitations of the study

Our study has several strengths. As far as we are aware, this is the first study focusing on top managers’ perceptions of WAM. Although work ability is already understood comprehensively, studies on management and work ability support practices have mainly approached the subject through disabilities. The study material was large enough and the same patterns started to repeat themselves referring to the saturation of the data. The studied organizations represented different geographical areas and the interviewees different fields of social and health care in Finland. Our research group was interdisciplinary and reflected all findings from different point of views and had ongoing conversations to gain a mutual understanding in case of disagreements. The accuracy of illustrative quotations’ translation from Finnish to English was approved collectively.

Some limitations of the study are also worth mentioning. First, the interviews were originally conducted for the development work of the “Strategic work ability management in the social and health care reform” project. This has partly guided the interview questions. Second, the project actors of the participating organizations provided the list of top managers who could be asked to participate in the study. Therefore, it is not possible to exclude the possibility that the sample was biased. However, to achieve credibility, it is important to find participants who have experiences of the phenomenon. Third, two out of sixteen interviews were conducted online due to the COVID19-pandemic. For the interviewees it may have been more difficult to express feelings online. During the online interviews the cameras were open, but as the interviews were not recorded the researchers were not able to afterwards return to the interview situation to e.g., read body language and interpret possible non-verbal communication. However, the online interviews may be more informal and relaxed as the interviewees are able to be at their homes making them feel more comfortable to share their thoughts [44]. Fourth, the combination of group and individual interviews was chosen mainly due to limited resources and time frame in the participated organizations. The individual interviews may contain more information as one may have more time to speak than in group interview. On the other hand, group interviews may encourage to discuss more critically or express more personal opinions [45]. There seemed to be a psychological safety in the group interviews, and everyone were able to share their thoughts. Finally, the organizations involved in the project, as well as the top managers interviewed, may have had more than average interest in work ability issues and have more critical thoughts about these matters.

## 5 Conclusions

Top managers’ multidimensional perception of WAM is crucial as it may influence the organizations’ work ability supporting measures. Adding proactive actions alongside reacting to problems, considering both individual and organizational level actions, and setting strategic level aims for WAM is essential to guarantee labor availability and personnel retention. This requires understanding and commitment of the organizations’ top management to manage work ability at a strategic level, especially during constant changes.

## References

[1] Ilmarinen J . From work ability research to implementation, International Journal of Environmental Research and Public Health 2019;16:2882. 31409037 10.3390/ijerph16162882 PMC6720430

[2] Loisel P . Côté P . The Work Disability Paradigm and Its Public Health Implications, In: Loisel P, Anema JR, editors. Handbook of work disability: Prevention and management. New York: Springer, 2013:59–67.

[3] Lederer V . Loisel P . Rivard M . Champagne F . Exploring the diversity of conceptualizations of work (dis)ability: A scoping review of published definitions, Journal of Occupational Rehabilitation 2014;24(2):242–67. 23884716 10.1007/s10926-013-9459-4

[4] Cadiz DM . Brady G . Rineer JR . Truxillo DM . A review and synthesis of the work ability literature, Work, Aging and Retirement 2019;5(1):114–38.

[5] Brady GM . Truxillo DM . Cadiz DM . Rineer JR . Caughlin DE . Bodner T . Opening the black box: Examining the nomological network of work ability and its role in organizational research, Journal of Applied Psychology 2020;105(6):637–70. 31647249 10.1037/apl0000454

[6] Shaw WS . Main CJ . Pransky G . Nicholas MK . Anema JR . Linton SJ . Employer policies and practices to manage and prevent disability: Foreword to the special issue, Journal of Occupational Rehabilitation 2016;26(4):394–8. 27562584 10.1007/s10926-016-9658-x PMC5104772

[7] Lerner D . Rodday AM . Cohen JT . Rogers WH . A systematic review of the evidence concerning the economic impact of employee-focused health promotion and wellness programs, Journal of Occupational & Environmental Medicine 2013;55(2):209–22. 23287723 10.1097/JOM.0b013e3182728d3c

[8] Reiman A . Ahonen G . Juvonen-Posti P . Heusala T . Takala EP . Joensuu M . Economic impacts of workplace disability management in a public enterprise, International Journal of Public Sector Performance Management 2017;3(3):297.

[9] Leino T . Turunen JKA . Pehkonen I . Juvonen-Posti P . Important collaborative conditions for successful economic outcomes of work disability management: A mixed methods multiple case study. Work. 2023;74(2):685–697. 36278370 10.3233/WOR-210026

[10] Camisa V . Gilardi F . Di Brino E . Santoro A . Vinci MR . Sannino S . et al. Return on investment (Roi) and development of a workplace disability management program in a hospital - a pilot evaluation study, International Journal of Environmental Research and Public Health 2020;17(21):8084. 33147861 10.3390/ijerph17218084 PMC7662934

[11] Mehta AJ . Mathisen J . Nguyen TL . Rugulies R . Hulvej Rod N . Chronic disorders, work-unit leadership quality and long-term sickness absence among 33 025 public hospital employees, Scandinavian Journal of Work, Environment and Health 2022;48(7):560–8. 10.5271/sjweh.4036 PMC10539112 35700335

[12] Pieper C . Schröer S . Eilerts AL . Evidence of workplace interventions - a systematic review of systematic reviews, International Journal Environmental Research and Public Health 2019;16(19):3553. 10.3390/ijerph16193553 PMC6801553 31547516

[13] Bryson J . George B . Strategic management in public administration, Subject: Policy, administration, and bureaucracy. Oxford Research Encyclopedia of Politics 2020.

[14] Hambrick DC . Mason PA . Upper echelons: The organization as a reflection of its top managers, The Academy of Management Review 1984;9(2):193.

[15] Gfrerer A . Hutter K . Füller J . Ströhle T . Ready or not: Managers’ and employees’ different perceptions of digital readiness, California Management Review 2021;63(2):23–48.

[16] Turner LA . Merriman KK . Cultural intelligence and establishment of organisational diversity management practices: An upper echelons perspective, Human Resource Management Journal 2022;32(2):321–40.

[17] Jasper M . Crossan F . What is strategic management? Journal of Nursing Management 2012;20(7):838–46. 10.1111/jonm.12001 23050617

[18] Kramar R . Sustainable human resource management: Six defining characteristics, Asia Pacific Journal of Human Resources 2022;60(1):146–70.

[19] Mjaku G . Strategic management and strategic leadershiInternational Journal of Scientific and Research Publications 2020;10(8):914–8.

[20] Nilsson K . Nilsson E . Organisational measures and strategies for a healthy and sustainable extended working life and employability— a deductive content analysis with data including employees, first line managers, trade union representatives and hr-practitioners, International Journal of Environmental Research and Public Health 2021;18(11):5626. 34070299 10.3390/ijerph18115626 PMC8197545

[21] Finnish Government. Health and social services reform [Internet] [accessed 2023 Jun 27], Available from: https://soteuudistus.fi/en/frontpage.

[22] Figueroa CA . Harrison R . Chauhan A . Meyer L . Priorities and challenges for health leadership and workforce management globally: A rapid review, BMC Health Services Research 2019;19(1):239. 31014349 10.1186/s12913-019-4080-7 PMC6480808

[23] The Finnish Institute of Occupational Health. Strategic work ability management in the social and health care reform [Internet] [accessed 2023 Jun 27]. Available from: https://www.ttl.fi/en/research/projects/strategic-work-ability-management-social-and-health-care-reform.

[24] Haukka E . Horppu R . Pehkonen I . Anttilainen J . Juvonen-Posti P . Bergbom B . et al. Strategic work ability management in the social and health care reform: Evaluation of the development project [Internet], Finnish Institute of Occupational Health. [accessed Jun 27]. Available from 2023. https://www.julkari.fi/handle/10024/145326.

[25] Pehkonen I . Turunen J . Juvonen-Posti P . Henriksson L . Vihtonen T . Seppänen J . et al. Collaboration in the successful work disability management, Multi-data and Mixed Method Research. [Internet]. [accessed 2023 Jun 27]. Available from: https://www.julkari.fi/handle/10024/132028.

[26] Tuisku K . Joutsenniemi K . Rentto T . Heikinheimo S . Resource-oriented assessment of work ability in psychiatry, Psychiatria Fennica 2015;46:125–45.

[27] Centre for Research on Work Disability Policy. The CSA Work Disability Management System Standard (CSA Zisnow available | CRWDP [Internet] [accessed Jun 27], Available from 2023. https://www.crwdca/en/node/753.

[28] Jansson I . Björklund A . Perseius KI . Gunnarsson AB . The concept of ‘work ability’ from the view point of employers, WORK 2015;52(1):153–67. 26410230 10.3233/WOR-152037

[29] Graebner ME . Martin JA . Roundy PT . Qualitative data: Cooking without a recipe, Strategic Organization 2012;10(3):276–84.

[30] Patton MQ . Qualitative research & evaluation methods: Integrating theory and practice, Fourth edition. Thousand Oaks, California: SAGE Publications, Inc 2015;64–117.

[31] Teherani A . Martimianakis T . Stenfors-Hayes T . Wadhwa A . Varpio L . Choosing a qualitative research approach, Journal of Graduate Medical Education 2015;7(4):669–70. 26692985 10.4300/JGME-D-15-00414.1 PMC4675428

[32] Graneheim UH . Lindgren BM . Lundman B . Methodological challenges in qualitative content analysis: A discussion paper, Nurse Education Today 2017;56:29–34. 28651100 10.1016/j.nedt.2017.06.002

[33] Braun V . Clarke V . Using thematic analysis in psychology, Qualitative Research in Psychology 2006;3(2):77–101.

[34] Nowell LS . Norris JM . White DE . Moules NJ . Thematic analysis: Striving to meet the trustworthiness criteria, International Journal of Qualitative Methods 2017;16(1):160940691773384.

[35] Braun V . Clarke V . What can “thematic analysis” offer health and wellbeing researchers? International Journal of Qualitative Studies on Health and Well-Being 2014;9(1):26152. 10.3402/qhw.v9.26152 PMC4201665 25326092

[36] Noble H . Smith J . Issues of validity and reliability in qualitative research, Evid Based Nurs 2015;18(2):34–5. 25653237 10.1136/eb-2015-102054

[37] Main CJ . Shaw WS . Employer policies and practices to manage and prevent disability: Conclusion to the special issue, Journal of Occupational Rehabilitation 2016;26(4):490–8. 27475446 10.1007/s10926-016-9655-0 PMC5104791

[38] Nilsson K . Nilsson E . Managers’ attitudes to different action proposals in the direction to extended working life: A cross-sectional study, Sustainability 2022;14(4):2182.

[39] Aura O . Ahonen G . Ilmarinen J . Strategic wellness management in Finland: The first national survey of the management of employee well-being, Journal of Occupational & Environmental Medicine 2010;52(12):1249–54. 21750473 10.1097/JOM.0b013e3181f75f90

[40] Julnes SG . Myrvang T . Reitan LS . Rønning G . Vatne S . Nurse leaders’ experiences of professional responsibility towards developing nursing competence in general wards: A qualitative study, Journal of Nursing Management 2022;30(7):2743–50. 35861024 10.1111/jonm.13745 PMC10087392

[41] Eriksson A . Axelsson R . Axelsson SB . Health promoting leadership – Different views of the concept, WORK 2011;40(1):75–84. 21849750 10.3233/WOR-2011-1208

[42] Montano D . Hoven H . Siegrist J . Effects of organisational-level interventions at work on employees’ health: A systematic review, BMC Public Health 2014;14(1):135. 24507447 10.1186/1471-2458-14-135 PMC3929163

[43] Vainieri M . Ferrè F . Giacomelli G . Nuti S . Explaining performance in health care: How and when top management competencies make the difference, Health Care Management Review 2019;44(4):306–17. 28448307 10.1097/HMR.0000000000000164 PMC6749958

[44] Moran L . Caetano A . Biographical research through the looking glass of social distancing: Reflections on biographical interviewing and online technologies in pandemic times, Irish Journal of Sociology 2022;30(2):209–13.

[45] Guest G . Namey E . Taylor J . Eley N . McKenna K . Comparing focus groups and individual interviews: Findings from a randomized study, International Journal of Social Research Methodology 2017;20(6):693–708.

